# Association between dental caries and adverse pregnancy outcomes

**DOI:** 10.1038/s41598-020-62306-2

**Published:** 2020-03-24

**Authors:** Geum Joon Cho, So-youn Kim, Hoi Chang Lee, Ho Yeon Kim, Kyu-Min Lee, Sung Won Han, Min-Jeong Oh

**Affiliations:** 1 Department of Obstetrics and Gynecology, Korea University College of Medicine, Seoul, Republic of Korea; 2 Department of Obstetrics and Gynecology, Feinberg School of Medicine, Northwestern University, Chicago, Illinois 60611 USA; 3 School of Industrial Management Engineering, Korea University, Seoul, Republic of Korea

**Keywords:** Epidemiology, Risk factors

## Abstract

Poor oral health is not only associated with diabetes and cardiovascular disease but adverse pregnancy outcomes. However the influence of dental caries on pregnancy is unknown. The aim of this study was to evaluate the association between dental caries and adverse pregnancy outcomes and the effect of treatment for dental caries on adverse pregnancy outcomes. Primiparas who delivered a singleton between January 1, 2010 and December 31, 2014 and underwent both general health examination and oral health examination during a National Korea Health Screening Examination within 1 year of pregnancy were eligible. The data of the women who met the inclusion criteria were linked to the data of their offspring contained within the National Korea Health Screening Program for Infants and Children database. Among 120,622 women who delivered during the study period, 28,623 (23.7%) women had dental caries. Among them, 4,741 (16.6%) women were treated for dental caries after diagnosis. In a multivariable analysis, women with dental caries had an increased risk of delivering large-for-gestational-age infants (odds ratio, 1.15; 95% confidence interval, 1.07, 1.23) compared to those without dental caries. When women with dental caries were divided on the basis of the treatment of dental caries, women with dental caries but no treatment had an increased risk of delivering large-for-gestational-age infants (odds ratio, 1.15; 95% confidence interval, 1.06, 1.24); conversely, there was no increased risk in women with dental caries and treatment compared with those without. Dental caries and its treatment were not associated with preterm birth and preeclampsia. Untreated dental caries was not associated with preterm birth or preeclampsia but with the risk of delivering large-for-gestational-age infants. These whole observation may be attributed to the various characteristics of mothers who develop dental caries are not treated.

## Introduction

Dental caries, defined as localized demineralization of hard tissues of the teeth by acid derived from food debris or sugar^1^, is one of the major oral health problems. The World Health Organization reported that approximately 2.4 billion individuals worldwide (35.3% of the total population) have dental caries^2^. Because of an increased craving for sweet and fast foods^3^, changes in oral factors, such as increased acidity in the mouth/saliva and reduction in saliva production, and fear of dental treatment^4,5^, pregnant women become more susceptible to^6^ and have a high prevalence of dental caries^7,8^.

Poor oral health is not only confined to the oral cavity but is also associated with systemic conditions, including diabetes and cardiovascular disease^9,10^. Increasing evidence suggests that poor oral health the inflammatory changes seen during periodontal disease may have adverse effect on pregnancy beyond the oral cavity. It has been reported that pregnant women with periodontal diseases have an increased risk of preterm birth, preeclampsia, and delivering small-for-gestational-age (SGA) infants^11,12^. Similarly, several studies have reported a positive association between dental caries and adverse pregnancy outcomes, including preterm birth and preeclampsia^13–16^. However, these findings have been challenged by further studies^8,17^. Moreover, the effectiveness of therapeutic interventions for periodontal diseases on the reduction of adverse pregnancy outcomes is inconsistent^18–20^. Thus, the aim of this study was to evaluate the association between dental caries and adverse pregnancy outcomes and the effect of treatment for dental caries on adverse pregnancy outcomes.

## Materials and methods

### Characteristics of the study dataset

This study was conducted by merging the Korea National Health Insurance (KNHI) claims database, National Health Screening Examination (NHSE), and National Health Screening Program for Infants and Children (NHSP-IC).

In Korea, 97% of the population is enrolled into the KNHI program. The KNHI claims database contains all claims information for these individuals. Therefore, nearly all information on the incidence of diseases and their treatments can be obtained from this centralized database, with the exception of procedures that are not covered by insurance, such as cosmetic surgery. Using the KNHI claims database, we identified all primiparas who delivered singletons between January 1, 2010 and December 31, 2014.

As a part of the KNHI system, all insurance subscribers and dependents were invited to participate in an NHSE free of charge. The NHSE consists of a general health examination and oral health examination. The pre-pregnancy characteristics of the study population were evaluated via a general health examination of the NHSE data. The presence or absence of dental caries in the study population was identified using an oral health examination of the NHSE data and then, through a link with the KNHI claims database, it was confirmed whether the women with caries had received treatment for dental caries before delivery.

The KNHI system also provides an NHSP-IC for all neonates for seven consecutive health examinations based on age groups (4 to 9 months, 9 to 18 months, 18 to 30 months, 30 to 42 months, 42 to 54 months, 54 to 66 months, and 66 to 80 months). The NHSP-IC consists of two components: a health interview with the parents and a health examination, including physical examination, anthropometric examination, and developmental screening of the offspring. Information on gestational age at delivery and birth weight was obtained through a health interview data conducted in the NHSP-IC.

### Study population

**Figure 1:**
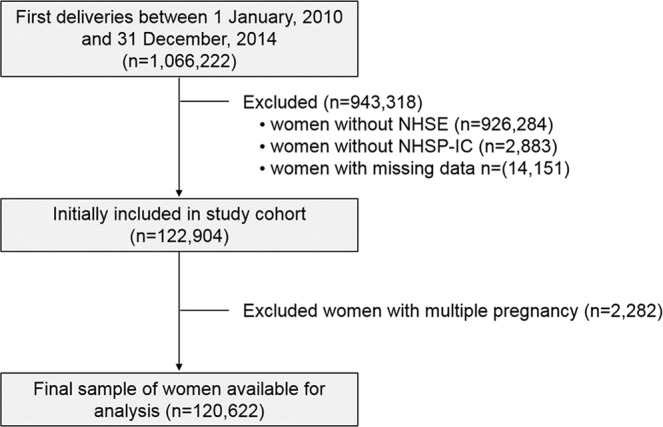
Flowchart of the enrollment of study participants. NHSE; National Health Screening Examination, NHSP-IC; National Health-Screening Program for Infants and Children.

Figure 1 illustrates the inclusion and exclusion criteria for the study participants. Using the KNHI claims database, we identified all mothers who had a delivery between January 1, 2010 and December 31, 2014. Mothers who delivered a live singleton infant and underwent both general health examination and oral health examination of an NHSE within 1 year of pregnancy were eligible. Of them, women whose offspring did not receive the NHSP-IC for the evaluation of the neonatal characteristics were excluded in the analysis. The study protocol was approved by the Institutional Review Board of Korea University Medical Center(2018GR0174). The IRB of Korea University Medical Center waived the need for informed consent because informed consents were unable to obtain due to deidentified centralized database from KNHI.

### Outcomes

In this study, various adverse pregnancy outcomes were confirmed. Using the KNHI claims dataset, preeclampsia was identified in accordance with the ICD-10 code. Data regarding gestational age at delivery and birth weight were abstracted from the NHSP-IC data. Preterm birth, low birth weight (LBW), and large for gestational age (LGA) were also identified. Preterm birth was defined as a gestational age of <37 weeks. LBW and LGA were defined as a birth weight of <2.5 kg and >4.0 kg, respectively.

### Measurement of pre-pregnancy characteristics

Pre-pregnancy maternal characteristics were identified through the general health examination of the NHSE data. Body mass index (BMI, in kg/m^2^) was calculated using the height and weight. Obesity was defined as a BMI of ≥25 kg/m^2^, which was adopted from the cutoffs proposed by the Korean Society for the Study of Obesity. Waist circumference was measured at the narrowest point between the lower border of the rib cage and the iliac crest during minimal respiration. Blood pressure (BP) was measured using a standard mercury sphygmomanometer. The levels of fasting glucose and total cholesterol (TC) were measured after a fast of at least 8 hours.

### Statistical analysis

Continuous and categorical variables were expressed as means ± standard deviations and percentages, respectively. Clinical characteristics were compared using the t-test or ANOVA with Duncan’s post hoc test for continuous variables and the chi-square test for categorical variables. Multivariable logistic regression analysis was used to estimate the adjusted odds ratios (ORs) and the 95% confidence intervals (CIs) for the association of dental caries with adverse pregnancy outcomes. All tests were two-sided, and p < 0.05 was considered statistically significant. Statistical analyses were performed using SAS for Windows, version 9.4 (SAS Inc., Cary, NC, USA).

## Results

Among 120,622 women who delivered during the study period, 28,623 (23.7%) women had dental caries. Among them, 4,741 (16.6%) women were treated for dental caries after diagnosis. Table 1 shows the basic characteristics of the participants according to the presence or absence of dental caries and the treatment of dental caries. Women with dental caries tended to be young and had high BMI, waist circumference, BP, fasting glucose level, and TC level compared with those without dental caries. The prevalence of obesity was higher in women with dental caries than in those without. When women with dental caries were divided on the basis of the treatment of dental caries, those with dental caries but no treatment had the highest prevalence of obesity. BMI, waist circumference, BP, and fasting glucose level were higher in women with dental caries without treatment compared to dental caries with treatment and no dental caries.

**Table 1:** Basic characteristics of the study population according to the presence or absence of dental caries and its treatment.

	No caries (n = 91,999)	Caries (n = 28,623)			p-value*	p-value^†^
		Total	with treatment (n = 4,741)	without treatment (n = 23,882)		
Age (years)	30.41 ± 3.31^a^	29.91 ± 3.53	29.73 ± 3.46^b^	29.94 ± 3.54^c^	<0.01	<0.01
BMI (kg/m^2^)	20.59 ± 2.59^a^	21.00 ± 2.98	20.77 ± 2.74^b^	21.05 ± 3.03^c^	<0.01	<0.01
Obesity (%)	5.95^a^	9.28	7.26^b^	9.68^c^	<0.01	<0.01
WC (cm)	68.60 ± 6.75^a^	69.47 ± 7.54	68.91 ± 7.02^b^	69.58 ± 7.64^c^	<0.01	<0.01
SBP (mmHg)	109.4 ± 10.64^a^	110.4 ± 10.93	109.98 ± 10.74^b^	110.53 ± 10.97^c^	<0.01	<0.01
DBP (mmHg)	68.95 ± 7.85^a^	69.65 ± 8.10	69.30 ± 7.97^b^	69.72 ± 8.12^c^	<0.01	<0.01
Fasting glucose level (mg/dL)	86.93 ± 10.06^a^	87.44 ± 11.61	86.86 ± 9.78^a^	87.56 ± 11.93^b^	<0.01	<0.01
Total cholesterol level (mg/dL)	176.9 ± 28.42^a^	176.5 ± 28.88	175.54 ± 28.10^b^	176.67 ± 20.03^a^	0.02	<0.01

*p-value for the no dental caries group vs the total dental caries group; ^†^p-value for the no dental caries group vs the dental caries with treatment group vs the dental caries without treatment group. Groups with the different superscripted letters (a.b and c) differed (p < 0.05 using Duncan’s post hoc test). Obesity, BMI ≥ 25 kg/m^2^ BMI, body mass index; WC, waist circumference; SBP, systolic blood pressure; DBP, diastolic blood pressure.

Table 2 shows the pregnancy outcomes between the groups. The infants of women with dental caries had a higher birth weight and prevalence of LBW and LGA than the infants of those without dental caries. However, the prevalence of preterm birth and preeclampsia was not different between the two groups. When women with dental caries were divided on the basis of the treatment of dental caries, those with dental caries but no treatment had the highest prevalence of delivering LGA infants; conversely, those with dental caries and treatment had the highest prevalence of delivering LBW infants among the three groups.

**Table 2:** Pregnancy outcomes of the study population according to the presence or absence of dental caries and its treatment.

	No dental caries (n = 91,999)	Dental caries			p-value*	p-value^†^
		Total (n = 28,626)	with treatment (n = 4,741)	without treatment (n = 23,882)		
Preterm birth (%)	1.78	1.73	2.02	1.67	0.56	0.19
Preeclampsia (%)	2.01	2.13	1.92	2.18	0.21	0.23
Birth weight (kg)	3.20 ± 0.45^a^	3.22 ± 0.47	3.21 ± 0.47^ab^	3.22 ± 0.47^b^	<0.01	<0.01
LBW infant delivery (%)	3.55^a^	3.65	3.88^b^	3.61^a^	<0.01	<0.01
LGA infant delivery (%)	3.15^a^	3.78	3.67^b^	3.80^c^	<0.01	<0.01

*p-value for the no dental caries group vs the total dental caries group; ^†^p-value for the no dental caries group vs the dental caries with treatment group vs the dental caries without treatment group. Groups with the different superscripted letters (a.b and c) differed (p < 0.05 using Duncan’s post hoc test).

LBW, low-birth-weight; LGA, large-for-gestational-age.

Table 3 shows the risk of developing adverse pregnancy outcomes according to the presence or absence of dental caries. In the multivariable logistic regression analysis, women with dental caries had an increased risk of delivering LGA infants (OR, 1.15; 95% CI, 1.07, 1.23). However, the other outcomes, including preterm birth, preeclampsia, and LBW infant delivery, were not different between the two groups.

**Table 3:** Risk of adverse pregnancy outcomes according to the presence or absence of dental caries and its treatment.

	Adjusted OR* (95% CI)			
	Preterm birth	Preeclampsia	LBW infant delivery	LGA infant delivery
Dental caries	0.97 (0.88, 1.08)	0.96 (0.88, 1.06)	1.04 (0.97, 1.12)	1.15 (1.07, 1.23)

*Adjusted for age, obesity, waist circumference, blood pressure, fasting glucose level, and total cholesterol level.

OR, odds ratio; CI, confidence interval; LBW, low-birth-weight; LGA, large-for-gestational-age.

When women with dental caries were divided on the basis of the treatment of dental caries (Table 4), those with dental caries but no treatment had an increased risk of delivering LGA infants compared with those without (OR, 1.15; 95% CI, 1.06, 1.24) after adjustment for confounding factors. However, there was no difference in the risk of delivering LGA infants between women with dental caries and dental treatment and those without dental caries (OR, 1.15; 95% CI, 0.99, 1.35).

**Table 4:** Risk of adverse pregnancy outcomes according to the presence or absence of dental caries and its treatment.

	Adjusted OR* (95% CI)			
	Preterm birth	Preeclampsia	LBW infant delivery	LGA infant delivery
No dental caries	1	1	1	1
Dental caries with treatment	1.17 (0.95, 1.44)	0.94 (0.76, 1.17)	1.13 (0.97, 1.31)	1.15 (0.99, 1.35)
Dental caries without treatment	0.93 (0.84, 1.04)	0.97 (0.87, 1.07)	1.03 (0.95, 1.11)	1.15 (1.06, 1.24)

*Adjusted for age, obesity, waist circumference, blood pressure, fasting glucose level, and total cholesterol level.

OR, odds ratio; CI, confidence interval; LBW, low-birth-weight; LGA, large-for-gestational-age.

## Discussion

In this study, we found that women with dental caries had a slightly but significantly increased risk of delivering LGA infants compared with women without dental caries. These results are in contrast with those from a study reporting the association between periodontal disease and SGA infant delivery^21^. Although the mechanism underlying the increased risk of delivering LGA infants in mothers with dental caries is not understood, this association may be because of the characteristics of women with dental caries. For example, sedentary lifestyle and eating and drinking habits are closely associated with being overweight and oral conditions^22^. Overweight adults are physically less active, eat more frequently, eat sweets every day, and prefer fast food over fruit and vegetables than normal-weight young adults^23^. Compared with women without dental caries in this study, women with dental caries had a high prevalence of obesity and high level of fasting glucose, which have been known to be risk factors for dental caries^24–27^ and LGA infant delivery^28,29^. Therefore, the risk of delivering LGA infants may be attributed to the various characteristics of mothers who develop dental caries. However, even after adjustment for factors, such as obesity and fasting glucose level, the women with dental caries in this study had an increased risk of delivering LGA infants. Therefore, it is necessary to evaluate other maternal characteristics such as eating behavior that are not analyzed in this study to understand the mechanism.

The effects of dental caries treatment on pregnancy outcomes were also analyzed in this study. Interestingly, when we divided the study participants into groups based on the treatment of dental caries, we found that the risk of delivering LGA infants increased only in the untreated group. As shown in this study, the prevalence of obesity and fasting glucose levels, risk factors for dental caries and LGA infant delivery, were the highest in the untreated group. Moreover, it has been reported that dental visits and services were less frequent in patients with obesity^30^ and diabetes and prediabetes^31^, which are high risk factors of dental caries and LGA infant delivery. Therefore, the characteristics of those mothers seems to be associated with LGA infant delivery rather than the effects of dental caries carries treatment.

There seems to inversely proportional relationship between LGA and LBW infants from women with dental caries. Although the mechanism underlying the inversely proportional relationship is not understood, this association may be related to the pregnancy outcomes. In this study, women with dental caries had a higher birthweight with the highest in the untreated group compared with those without dental caries. Thus, while the prevalence of LGA increases, the prevalence of LBW decreases, which seems to have an inverse relationship.

Preterm birth and preeclampsia are known to be associated with maternal inflammatory complications^11^. Several studies have reported the association of periodontal diseases with preterm birth and preeclampsia^11,12,22^. *Streptococcus mutans* is the primary etiological agent of human dental caries^32^. Thus, untreated dental caries may result in further inflammatory complications^33,34^, which could affect pregnancy outcomes^35^. Similarly, we hypothesized that dental caries may be associated with preterm birth and preeclampsia through an infectious process but found that there was no association among them, which is inconsistent with the results from other studies^13–16^. Furthermore, we did not find any significant difference according to the treatment for dental caries.

The consequences of overall oral health, including that in pregnant women, are of great concern^36^. However, a large proportion of women in this study had dental caries and continued their pregnancy without treatment, which is consistent with the results from other studies^7,8^. Despite the increased risk of dental caries during pregnancy, they do not seem to take a routine check-up and treatment^5^. The results of this study confirm that dental caries is associated with LGA infant delivery and its treatment is associated with a reduction in risks. Women with LGA infants have a higher rate of cesarean sections; further, LGA neonates have an increased risk for birth trauma and shoulder dystocia and are more likely to develop obesity, diabetes, and cardiovascular diseases in their future life^37,38^. Otherwise, the dental caries and its treatment were not associated with other adverse pregnancy outcomes, such as preterm birth or preeclampsia. Therefore, our results indicate that if a woman is planning a pregnancy or is pregnant, regular check-ups and treatments for dental caries are needed without fear of dental treatment.

Several limitations should be considered when interpreting our findings. In this study, we evaluated the presence of dental caries by a dental examination; however, its number or severity of dental caries was not evaluated because these data were not available in this dataset. It has been reported that the number of dental caries was associated with an increased risk of preeclampsia^14^. Further studies are needed to evaluate the association between dental caries and adverse pregnancy outcomes considering its number and severity. In addition, there is no accurate result as to whether the dental caries has been cured because of the lack of information. Last, in this study, as only women who underwent both general health examination and oral health examination of an NHSE were included within 1 year of pregnancy, the presence or absence of dental caries in the study population could be identified accurately. Otherwise, since whether the women with caries had received treatment for dental caries before delivery was confirmed thorough a link with the KNHI claims database, there is no information about factors which may be prohibiting a portion of enrolled mothers to be not able to receive the dental treatment. Randomized controlled trials are required to confirm the effect of dental carries treatment on pregnancy outcomes.

Nevertheless, the strength of the present study lies in the evaluation of data from a large population-based study. To our knowledge, this is the study that included the largest number of subjects. Moreover, we evaluated the pre-pregnancy risk factors for dental caries, such as BMI and fasting glucose level, and confirmed whether the dental caries was treated to evaluate the effect of treatment on pregnancy outcomes.

In conclusion, dental caries was not associated with preterm birth or preeclampsia but with LGA infant delivery; its prevalence increased only in the untreated group. These whole observation may be attributed to the various characteristics of mothers who develop dental caries are not treated.
